# Supplementary material for: Quantitative assessment of fecal contamination in multiple environmental sample types in urban communities in Dhaka, Bangladesh using SaniPath microbial approach
Source: PLoS One. 2019 Dec 16;14(12):e0221193. doi: 10.1371/journal.pone.0221193 (PMC6913925; doi:10.1371/journal.pone.0221193)
Supplement: S1 Table — (DOC) [file pone.0221193.s001.doc]

| Pathway | Definitions |
| --- | --- |
| Municipal drinking water | Water supplied by the Water Supply and Sewerage Authority [WASA], including both “legal” and “illegal” connections. Water may be accessed through: piped water into compounds (including flexible pipes); public taps/standpoints (a formally designated water station in the community, provided by the government, or managed by someone in the community); or water vendors/trucks. All drinking water samples were collected directly from the source and not from household storage containers. |
| Non-municipal drinking water | Water that was not directly provided by WASA and was commonly used as drinking water in the neighborhood. These included shallow tube wells, submersible pump connected to deep tube wells, and 20 L commercially-available jars/bottles of water supplied by local vendor. |
| Bathing water | Water used most frequently for bathing by the children in the community. This included both municipal and non-municipal water supplies (i.e., municipal tapwater or stored municipal water, shallow tubewell water, or surface water). Bathing water was stored or used directly from the source, as reported by the users during sample collection. Bathing water was stored or used directly from the source, as reported by the users during sample collection. |
| Surface water | Water collected from lakes and ponds |
| Drain water | Water from a channel carrying liquid and solid waste, including rainwater, floodwater, and sewage |
| Floodwater | Stagnant water that remains for at least one hour after raining |
| Communal /shared latrine | Communal latrines are accessed by any neighborhood resident. Shared latrines are accessed only by specific households. These latrines are not located within a household. The fieldworkers collected latrine swabs from most frequently touched surfaces (walls and door lock/handles) of the selected latrines. |
| Raw produce | Vegetables that are commonly eaten without cooking. These vegetables do not have a shell or peel and grow above ground. Common produce items include cucumber, tomato, and coriander. |
| Street food [15,28] | Food sold on the street and commonly eaten by people in the community. Common street food we collected included *fuska* (a round puffed and fried crisp; a hole is created on the top to add a spiced sauce filling), *Chotpoti* (popular hot and sour snacks among the urban people in Bangladesh which is made up of [potatoes](https://en.wikipedia.org/wiki/Potato), [chickpeas](https://en.wikipedia.org/wiki/Chickpea), [onions](https://en.wikipedia.org/wiki/Onion) and [chilies](https://en.wikipedia.org/wiki/Chillies) mixed with tamarind sauce), and *Jhalmuri* (mixture of puffed rice and a variety of spices, including peanuts, mustard oil, chili, onion, tomato, fresh ginger, salt, and/or lemon juice). |
| Soil | Soil/sand/mud was collected and analyzed from areas where people gather and/or where children play within the neighborhood. |
