# Supplementary material for: Quantitative assessment of fecal contamination in multiple environmental sample types in urban communities in Dhaka, Bangladesh using SaniPath microbial approach
Source: PLoS One. 2019 Dec 16;14(12):e0221193. doi: 10.1371/journal.pone.0221193 (PMC6913925; doi:10.1371/journal.pone.0221193)
Supplement: S2 Table — *For Example: 1:100 dilution mean 1 unit of sample added with 99 units of PBS. (DOC) [file pone.0221193.s002.doc]

# Supporting information

| **Sample type** | **Undiluted** | **1:10** | **1:100*** | **1:1000** | **1:10,000** | **1:100,000** | **1:1,000,000** |
| --- | --- | --- | --- | --- | --- | --- | --- |
| **Drinking Water** | √ | √ |  |  |  |  |  |
| **Bathing Water** | √ | √ |  |  |  |  |  |
| **Surface Water** |  |  | √ | √ | √ |  |  |
| **Drain Water** |  |  |  |  |  | √ | √ |
| **Floodwater** |  |  | √ | √ | √ |  |  |
| **Produce** |  |  | √ | √ |  |  |  |
| **Street Food** |  | √ | √ | √ |  |  |  |
| **Latrine Swabs** |  | √ |  | √ |  |  |  |
| **Soil** |  |  | √ | √ | √ |  |  |
|  | | | | | | | |
