# Supplementary material for: Quantitative assessment of fecal contamination in multiple environmental sample types in urban communities in Dhaka, Bangladesh using SaniPath microbial approach
Source: PLoS One. 2019 Dec 16;14(12):e0221193. doi: 10.1371/journal.pone.0221193 (PMC6913925; doi:10.1371/journal.pone.0221193)
Supplement: S3 Table — (DOC) [file pone.0221193.s003.doc]

| Definition of neighborhoods | |
| --- | --- |
| Floating community: poorest quintile [23, 85] | Floating communities are made up of transient people who have no permanent dwelling units, and/or travelers from different parts of the country. These are the areas with the biggest bus terminals, railway stations and truck stands where thousands of people come every day and stay temporarily. (We selected Gabtoli bus stand and Kamalapur railway station areas as floating communities.) |
| Unstructured slum: poorest quintile [84] | A slum where most of the houses are constructed of very poor materials, such as walls and roofs made of straw, leaves, gunny sacks, polythene paper, bamboo, or tin. Most of the latrines are either hanging or made with bamboo, straw leaves, gunny sacks, and/or polythene paper. The majority of the water is supplied through flexible leaky pipes outside the compound or in the public space. (We selected Kalshi and Shampur areas as unstructured slum communities.) |
| Structured slum: second quintile [84] | A slum with moderately good household structures constructed from bricks and/or tin. Most of the households have access to shared piped drinking water within the compound and have relatively improved/shared latrine facilities compared to an unstructured slum. (We selected Badda and Hazaribagh areas as structured slum communities.) |
| Slum/low-income community: poorest and second quintiles [84] | A slum/low-income community is a cluster of compact settlements of five or more households that generally grows haphazardly and may be located on government or private vacant land. (For analysis, we combined both unstructured (Kalshi+Shampur) and structured slums (Badd+Hazaribagh) together for the category of slum/ low-income communities.) |
| High-income elite community: highest quintile [84] | These are the affluent [neighborhood](https://en.wikipedia.org/wiki/Neighbourhood)s in [Dhaka](https://en.wikipedia.org/wiki/Dhaka): a residential area for elites and home to a number of the city's restaurants, shopping centres, schools, and members' clubs. These neighborhoods also host the majority of [embassies](https://en.wikipedia.org/wiki/Embassy) and [high commissions](https://en.wikipedia.org/wiki/High_commission) in Dhaka. These areas include Gulshan, Baridhara, Banasree, Dhanmondi R/A, Bashundhara R/A, etc., and they attract upper-class residents. These areas also have improved WASH facilities compared to rest of the city. (We selected Gulshan and Dhanmondi areas as high-income elite communities) |
| Middle-income commercial/business area: middle quintile [84] | This is a major business and commercial hub of Dhaka city and has more offices and business institutions than any other part of the city. It is home to the largest number of corporate headquarters in the nation. Government officials also live in old apartments with relatively poor WASH facilities compared to high-income elite communities. Large numbers of public latrines are also available in these neighborhoods. (We selected AGB officers' colony, Motijhil area, as a middle-income commercial community.) |
| Middle-income newly developed area: middle and highest quintiles [84] | This is a newly-extended northern [Thana](https://en.wikipedia.org/wiki/Administrative_divisions_of_Bangladesh) (administrative sub district) and a planned square grid residential suburb of [Dhaka](https://en.wikipedia.org/wiki/Dhaka) which is geographically elevated from southern part of Dhaka. This newly-extended area has a relatively low population density, a good sewerage system and is less prone to flooding. (We selected the Uttarkhan area as the middle-income newly-extended community) |
| High-income community: both middle and highest quintiles [84] | For analysis, we combined high-income elite (Gulshan+ Dhanmondi), middle-income commercial/business community (Motijhil) and newly developed neighborhoods (Uttarkhan) together to define high-income communities. |
| Dhaka City Corporation [22]: | In the Local Govt. (City Corporation) Amendment Act (2011), Dhaka City Corporation (DCC) was divided and re-created as Dhaka South City Corporation (DSCC) and [Dhaka North City Corporation](https://en.wikipedia.org/wiki/Dhaka_North_City_Corporation) (DNCC) on 04.12.2011. |
