# Supplementary material for: Quantitative assessment of fecal contamination in multiple environmental sample types in urban communities in Dhaka, Bangladesh using SaniPath microbial approach
Source: PLoS One. 2019 Dec 16;14(12):e0221193. doi: 10.1371/journal.pone.0221193 (PMC6913925; doi:10.1371/journal.pone.0221193)
Supplement: S4 Table — Jhalmuri: mixture of puffed rice and a variety of spices, including peanuts, mustard oil, chili, onion, tomato, fresh ginger, salt and/or lemon juice Chotpoti: popular hot and sour snacks among the urban people in Bangladesh, which is made of potatoes, chickpeas, onions, and chilies mixed with tamarind sauce Fuska: a round, puffed and fried crisp; a hole is created on the top to add a spiced sauce filling Halim: made of wheat, barley, meat (usually minced beef or mutton), lentils and spices, sometimes rice is also used. Dal puri: an unleavened deep-fried bread along with lentils, onions and/or (rock) salt. (DOC) [file pone.0221193.s004.doc]

| **Characteristics** | Latrine type | Open drain present | Do Children play in/near the drain | Common bathing water source | Common municipal drinking water source | Common non-municipal drinking water source | Common surface water source | Common types of produce eaten raw | Common types of street food eaten | Does it flood in this neighborhood? | Do children play in the flood-water? |
| --- | --- | --- | --- | --- | --- | --- | --- | --- | --- | --- | --- |
| **Categories** | Private latrine (1), Shared latrine (2), Public latrine (3) | Yes (1)  No (0) | Yes (1)  No (0) | WASA (1), Shallow Tubewell (2), Surface water (3) | WASA tap (1), WASA handpump (2), WASA Public tap (3) | Jar water (1), Deep borewell (2), Submersible pump (3), Bottled water (4) | Ditch (1), Pond (2), Lake (3), River (4), Canal (5) | Cucumber (1), tomato (2), coriander (3), carrot (4), green chili (5), Mint (6), Capsicum (7) | *Jhalmuri*(1), *Chotpoti*(2), *Fuska* (3), *Halim* (4), *Dal puri* (5), Pickle (6) | Yes (1)  No (0) | Yes (1)  No (0) |
| **Neighborhoods** | |  |  |  |  |  |  |  |  |  |  |
| **Floating communities** | |  |  |  |  |  |  |  |  |  |  |
| Gabtoli | 2, 3 | 1 | 0 | 1, 3 | 1 | 1 | 1 | 1, 3, 4, 5, 6 | 1, 2, 3, 4 | 1 | 1 |
| Kamalapur | 2, 3 | 1 | 1 | 1, 2 | Nil | 1, 2 | 1, 2, 5 | 1, 2, 3, 4, 5 | 1, 2, 3 | 1 | 1 |
| **Unstructured slums** | |  |  |  |  |  |  |  |  |  |  |
| Kalshi | 2, 3 | 1 | 1 | 1, 2 | 1 | 1 | 3 | 1, 2, 4 | 1, 2, 3 | 1 | 1 |
| Shampur | 2 | 1 | 1 | 1, 2 | 1 | 1 | 2 | 1, 3, 4, 5 | 1, 3 | 1 | 1 |
| **Structured slums** | |  |  |  |  |  |  |  |  |  |  |
| Badda | 1, 2, 3 | 1 | 1 | 1, 2 | 1, 2 | 1 | 1 | 1, 3, 4, 5 | 1, 2, 3 | 1 | 1 |
| Hazaribag | 1, 2 | 1 | 1 | 1, 2 | 1, 2, 3 | 1 | 4, 5 | 1,2, 3, 4, 5 | 1, 2, 3, 5 | 1 | 1 |
| **Non-slum with poor WASH facilities** | | | |  |  |  |  |  |  |  |  |
| Uttarkhan | 1, 2, 3 | 1 | 0 | 1, 2 | Nil | 3 | 2 | 1,2, 3, 4, 5 | 1, 2, 3, 6 | 1 | 1 |
| Motijhil | 1, 2, 3 | 1 | 1 | 1 | 1, 3 | 1 | 2 | 1, 3, 4, 5 | 1, 2, 3, 4 | 1 | 1 |
| **Non-slum with improve WASH facilities** | | | |  |  |  |  |  |  |  |  |
| Gulshan | 1, 2, 3 | 1 | 0 | 1 | 1 | 1, 4 | 3 | 1, 2, 4, 7 | 1, 2, 3 | 0 | 0 |
| Dhanmondi | 1, 2, 3 | 0 | 0 | 1, 3 | 1 | 1, 4 | 3 | 1, 2, 4 7 | 1, 2, 3 | 1 | 1 |
