# Supplementary material for: Quantitative assessment of fecal contamination in multiple environmental sample types in urban communities in Dhaka, Bangladesh using SaniPath microbial approach
Source: PLoS One. 2019 Dec 16;14(12):e0221193. doi: 10.1371/journal.pone.0221193 (PMC6913925; doi:10.1371/journal.pone.0221193)
Supplement: S5 Table — (DOC) [file pone.0221193.s005.doc]

| **Characteristics** | **N=100** |
| --- | --- |
| **Latrines** | **%** |
| Latrine type |  |
| Piped to sewage system or elsewhere | 65 |
| Pit latrine with slab or water seal | 12 |
| Ventilated improved pit latrine | 12 |
| Open pit latrine | 5 |
| Flush to septic tank | 6 |
| Type of latrine (select one) |  |
| Shared | 100 |
| Number of toilets per block, Mean (SD) | 2.27 (1.67) |
| One | 37 |
| Two | 38 |
| 3-4 | 17 |
| 5-10 | 8 |
| Number of toilets in block with feces visible on walls and/or slabs | 31 |
| Number of toilet users per block per day, median (IQR) | 30 (15-63.5) |
| Handwashing station present near latrine | 85 |
| Level of *E. coli* in collected samples (MPN/swab) | |
| Nonedetected | 85 |
| 1-<10 | 12 |
| 10-100 | 3 |
| **Soil** |  |
| Type of Soil |  |
| Only soil | 11 |
| Only sand | 17 |
| Mixed | 72 |
| Weight (gram) of collected soil, median (IQR) | 10.2 (9.99-10.5) |
| Distance (meter) from closest latrine from the sample site (mean, SD) | 14.17 (7.47) |
| < 10 meters | 20 |
| 10-20 meters | 65 |
| >20 meters | 15 |
| Level of *E. coli* collected samples (MPN/gram) | |
| None detected | 11 |
| 1-<10 | 10 |
| 10-100 | 24 |
| >100 | 55 |
| Presence of feces within 3 meters of sampling area |  |
| Yes | 57 |
| No | 43 |
| **Drain** |  |
| Water turbidity (Nephelometric Turbidity Units [NTU]), mean (SD) | 120.1 (137.1) |
| <50 | 24 |
| 51-100 | 25 |
| >100 | 51 |
| Water level |  |
| Low (~1/4th full) | 52 |
| Medium (~1/2 full) | 29 |
| High (3/4th+ full) | 19 |
| Water flow |  |
| Flowing/Moving | 80 |
| Stagnant | 20 |
| Drain size |  |
| Small (<0.5 meters wide) | 42 |
| Medium (0.5-1 meters wide) | 41 |
| Large (1-3 meters wide) | 13 |
| Extra large (>3 meters wide) | 4 |
| Primary type of drain lining |  |
| Cement | 74 |
| Dirt/mud | 22 |
| Other | 4 |
| Level of *E.coli* in collected samples (MPN/100ml) | |
| <10,000 | 17 |
| >10,000 | 83 |
| **Bathing water** |  |
| Turbidity (NTU), mean (SD) | 1.11 (2.06) |
| <1 | 71 |
| 1-<5 | 24 |
| >5 | 5 |
| Free chlorine residual |  |
| <0.20 mg/L | 98 |
| >0.20 mg/L | 2 |
| Total chlorine residual |  |
| <0.20 mg/L | 96 |
| >0.20 mg/L | 4 |
| Source type of bathing water |  |
| Municipal supplied water | 73 |
| Tube well/borehole | 25 |
| Dug well | 2 |
| Water stored in a container | 25 |
| Water was covered | 15 |
| Level of *E. coli* in collected samples (MPN/100ml) | |
| None detected | 69 |
| 1-<10 | 18 |
| 10-100 | 10 |
| >100 | 3 |
| **Municipal supplied drinking water** |  |
| Turbidity (NTU), mean (SD) | 0.85 (1.33) |
| <1 | 74 |
| 1-<5 | 25 |
| >5 | 1 |
| Free chlorine residual |  |
| <0.20 mg/L | 97 |
| >0.20 mg/L | 3 |
| Total chlorine residual |  |
| <0.20 mg/L | 91 |
| >0.20 mg/L | 9 |
| Type of connection |  |
| Legal | 80 |
| Illegal | 20 |
| Source of water |  |
| Piped water into Compound | 72 |
| Deep Tube Well | 23 |
| Other | 4 |
| Level of *E. coli* in collected samples (MPN/100ml) |  |
| None detected | 69 |
| 1-<10 | 14 |
| 10-100 | 11 |
| >100 | 6 |
| **Non-municipal drinking water** |  |
| Type of source |  |
| 20 L Jar | 90 |
| Submersible pump | 10 |
| Turbidity (NTU), mean (SD) | 0.33 (1.14) |
| <1 | 93 |
| 1-<5 | 6 |
| >5 | 1 |
| Free chlorine residual, mean (SD) | 0.04 (.002) |
| <0.20 mg/L | 100 |
| Total chlorine residual | 0.10 (0.01) |
| <0.20 mg/L | 100 |
| Level of *E. coli* in collected samples (MPN/100ml) | |
| None detected | 95 |
| 1-<10 | 2 |
| 10-100 | 3 |
| >100 | 0 |
| **Surface water** |  |
| Turbidity (NTU), mean (SD) | 43.59 (47.50) |
| < 10 | 19 |
| 10-20 | 20 |
| >20 | 61 |
| Source of surface water |  |
| River | 3 |
| Lake or pond | 97 |
| Level of *E. coli* in collected samples (MPN/100ml) | |
| None detected | 0 |
| 1-<10 | 4 |
| 10-100 | 17 |
| >100 | 79 |
| **Produce** |  |
| Type of produce sampled |  |
| Tomato | 34 |
| Coriander | 31 |
| Cucumber | 35 |
| Weight (gram) of collected produce, median (IQR) | 129.6 (33.6-165.6) |
| Tomato | 132.6 (108.8-152.9) |
| Cucumber | 171.1 (12.8-745.2) |
| Coriander | 16.3 (10.9-35.4) |
| Level of *E. coli* in collected samples (MPN/serving) | |
| None detected | 43 |
| 1-<10 | 17 |
| 10-100 | 24 |
| >100 | 16 |
| Mean log_10_ MPN *E. coli* concentrations per serving (sd) |  |
| Tomato | 2.13 (0.91) |
| Cucumber | 2.94 (0.97) |
| Coriander | 4.64 (0.82) |
| Overall | 3.19 (1.36) |
| Mean log_10_ MPN *E. coli* concentrations per gram (sd) |  |
| Tomato | -0.02 (0.97) |
| Cucumber | 0.72 (1.08) |
| Coriander | 3.32 (0.84) |
| Overall | 1.27 (1.71) |
| **Street food** |  |
| Type of Street Food |  |
| Fuska/chotpoti | 62 |
| Jhalmuri | 38 |
| Weight (gram) of collected street food, median (IQR) | 105.7 (62.4-174.0) |
| Fuska/chotpoti | 145.2 (99.6-187.5) |
| Jhalmuri | 46.0 (40.2-88.2) |
| Level of *E. coli* in collected samples (MPN/gram) | |
| None detected | 27 |
| 1-<10 | 27 |
| 10-100 | 28 |
| >100 | 18 |
| **Flood water** |  |
| Turbidity (NTU), mean (SD) | 299.17 (263.1) |
| <50 | 13 |
| 51-100 | 22 |
| >100 | 65 |
| Distance from closest latrine |  |
| < 10 meter | 31 |
| 10-20 meter | 35 |
| >20 meter | 34 |
| Level of *E. coli* in collected samples (MPN/100ml) | |
| None detected | 1 |
| 1-<10 |  |
| 10-100 | 14 |
| >100 | 78 |
