# Supplementary figures and images for: Quantitative assessment of fecal contamination in multiple environmental sample types in urban communities in Dhaka, Bangladesh using SaniPath microbial approach
Source: PLoS One. 2019 Dec 16;14(12):e0221193. doi: 10.1371/journal.pone.0221193 (PMC6913925; doi:10.1371/journal.pone.0221193)

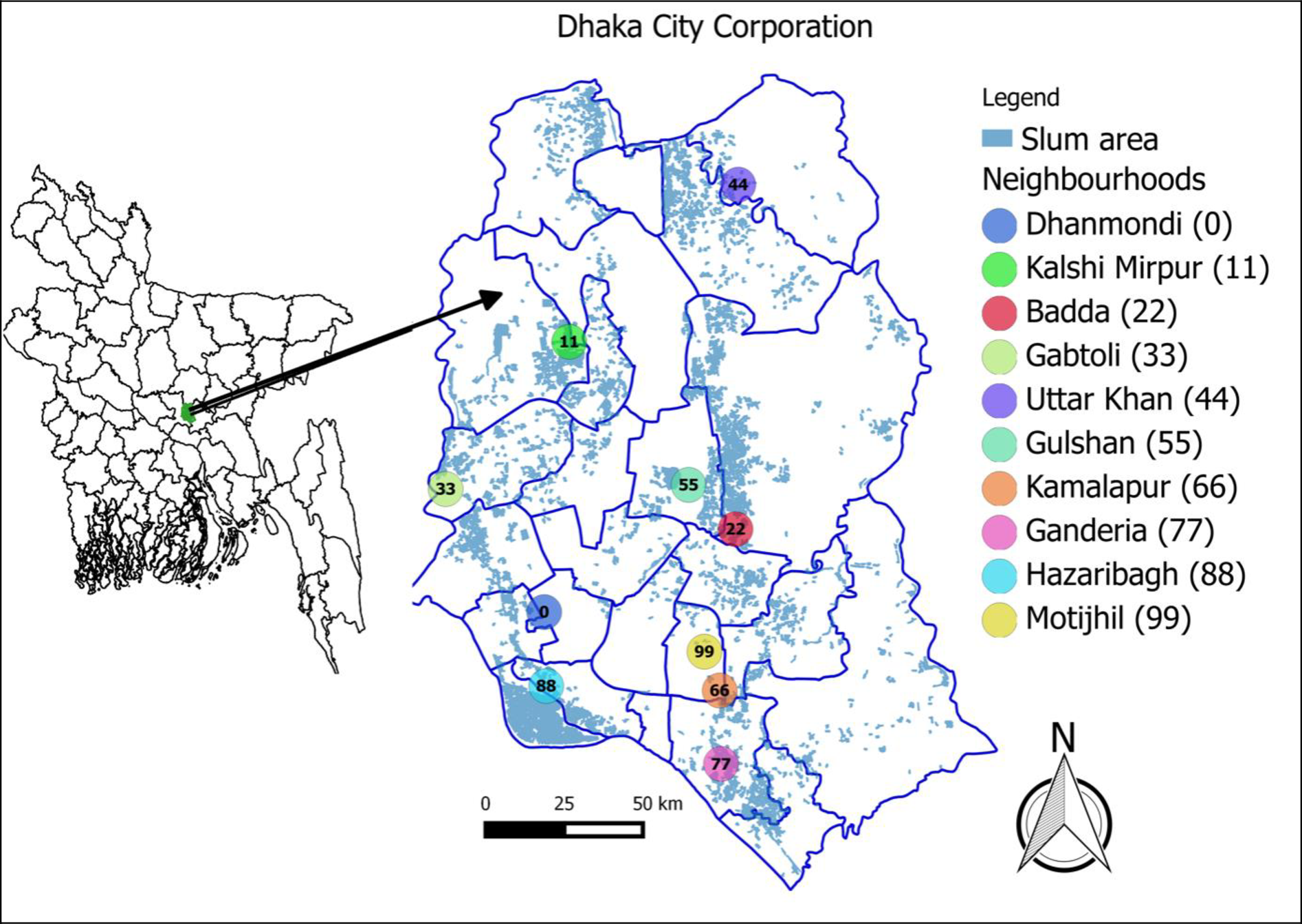

Supplement: S1 Fig — *Numbers within the map represent different neighborhood codes. (TIF) [file pone.0221193.s006.tif]
